# Identification and profiling of microRNAs and differentially expressed genes during anther development between a genetic male-sterile mutant and its wildtype cotton via high-throughput RNA sequencing

**DOI:** 10.1007/s00438-020-01656-y

**Published:** 2020-03-14

**Authors:** Dingwei Yu, Libei Li, Hengling Wei, Shuxun Yu

**Affiliations:** State Key Laboratory of Cotton Biology, Institute of Cotton Research, Chinese Academy of Agriculture Sciences (CAAS), Anyang, 455000 Henan People’s Republic of China

**Keywords:** Cotton (*gossypium hirsutum*), Genetic male sterility (GMS), microRNAs (miRNAs), Degradome, Transcriptome, Target gene

## Abstract

Genetic male sterility (GMS) facilitates hybrid seed production in crops including cotton (*Gossypium hirsutum*). However, the genetic and molecular mechanisms specifically involved in this developmental process are poorly understood. In this study, small RNA sequencing, degradome sequencing, and transcriptome sequencing were performed to analyze miRNAs and their target genes during anther development in a GMS mutant (‘Dong A’) and its fertile wildtype (WT). A total of 80 known and 220 novel miRNAs were identified, 71 of which showed differential expressions during anther development. A further degradome sequencing revealed a total of 117 candidate target genes cleaved by 16 known and 36 novel miRNAs. Based on RNA-seq, 24, 11, and 21 predicted target genes showed expression correlations with the corresponding miRNAs at the meiosis, tetrad and uninucleate stages, respectively. In addition, a large number of differentially expressed genes were identified, most of which were involved in sucrose and starch metabolism, carbohydrate metabolism, and plant hormone signal transduction based on Gene Ontology (GO) and Kyoto Encyclopedia of Genes and Genomes (KEGG) analysis. The results of our study provide valuable information for further functional investigations of the important miRNAs and target genes involved in genetic male sterility and advance our understanding of miRNA regulatory functions during cotton anther development.

**Electronic supplementary material:**

The online version of this article (10.1007/s00438-020-01656-y) contains supplementary material, which is available to authorized users.

## Introduction

Upland cotton (*Gossypium hirsutum*) is an important cultivated economic crop that provides the majority of natural textile fiber materials worldwide. Breeding cotton cultivars with high-fiber quality and lint yield has been critical to meet increased economic demand. The utilization of hybrid vigor is a key strategy for improving cotton yield and quality with higher effectiveness (Huang, Yang et al. 2016). Male sterility is a kind of universal phenomenon in plants and is considered the main way to produce hybrid seeds in cotton because of its simple and efficient pollination control system. Therefore, it is necessary for scientists to perform intensive investigations of the genetic and molecular mechanisms of male sterility.

Male sterility has been reported in a lot of plant species and can cause abnormal development of either the sporophytic or gametophytic anther tissues. Male sterility provides crucial breeding tools for harnessing hybrid vigor and provides important materials with which to study stamen and pollen development. According to the genetic mechanism, male sterility can be divided into cytoplasmic male sterility (CMS) and genetic male sterility (GMS). CMS is caused by mitochondrial genes with coupled nuclear genes, while GMS is caused by nuclear genes alone. Because most CMS systems have stringent restorers, cytoplasmic negative effects, and unstable sterility (Schnable and Wise 1998), the GMS system has attracted more attention from the cotton breeding community for cultivating hybrid cotton with high fiber quality and yield. Although many studies have focused on cotton GMS, the molecular mechanism specifically involved in this developmental process is still poorly understood.

MicroRNAs (miRNAs) are a distinct class of endogenous, noncoding small RNAs that play a very important role in posttranscriptional gene regulation via the degradation of target mRNAs or the repression of targeted gene translation (Voinnet 2009). Increasing evidence shows that miRNAs are vital components of the posttranscriptional regulation of gene expression, which is important for almost all aspects of plant biological development, such as tissue differentiation, signal transduction, growth phase switching, and response to stresses (Vance and Vaucheret 2001; Baulcombe 2004; Millar and Gubler 2005; Xie and Qi 2008; Li et al. 2011; Soitamo et al. 2012; Yang et al. 2013a, b, c; Cui et al. 2014; Kontra et al. 2016; Liu and Chen 2016; Adkar-Purushothama et al. 2018; Li and Wang 2018; Zhang et al. 2018,2019; Sun Sparks et al. 2019). Recently, with the rapid development of high-throughput sequencing technology, several studies have identified miRNAs and their target genes on a genome-wide level in crops during anther development (Chambers and Shuai 2009; Wei et al. 2013a, b; Yang et al. 2013a, b, c, 2016; Jiang et al. 2014; Zhang et al. 2016,2018). For example, a comparative miRNAome analysis was performed between CMS and maintainer lines in *Raphanus sativus*, and 28 known and 14 potential novel miRNAs were detected to be differentially expressed, revealing a putative regulatory network involved in anther development (Zhang et al. 2016). In addition, in *Brassica,* 54 known and 8 novel miRNAs were identified to be involved in pollen development by small RNA sequencing (Jiang et al. 2014).

To date, many studies have investigated miRNAs and their regulatory mechanisms in specific biological processes of cotton, such as somatic embryogenesis (Yang et al. 2013a, b, c), fiber development (Pang et al. 2009; Xue et al. 2013; Wang et al. 2016), and response to stresses (Xie et al. 2015). The miRNA expression profiles in the flower buds of a novel male-sterile cotton line, Yu98-8A, and its fertile wildtype (WT) revealed that 49 conserved and 51 novel miRNAs were differentially expressed, which indicated the regulatory complexity of miRNAs and their target genes in the regulation of cotton male sterility (Yang et al. 2016). A combined small RNA and transcriptome sequencing analysis in a three-line hybrid cotton system identified 10 and 30 differentially expressed miRNA–target gene pairs in floral buds and revealed putative regulatory roles during anther development in CMS and fertility restoration (Zhang et al. 2018).

For *G. hirsutum*, the GMS mutant (‘Dong A’) and its fertile WT are ideal genetic materials for studying cotton anther development and male sterility because of their shared genetic background. In our previous studies, a comparative small RNA sequencing and degradome analysis identified 16 conserved miRNA families, six of which were significantly differentially expressed at the three different stages of anther development between GMS (Dong A) and its WT (Wei et al. 2013a, b). In our other previous study, combined digital gene expression experiments and real-time quantitative RT-PCR (qRT-PCR) were also performed to reveal the expression of many key genes involved in cotton anther development (Wei et al. 2013a, b). However, at that time, due to the lack of a tetraploid upland cotton reference genome sequence, the data were not fully utilized. With the goal of systematically screening and identifying potential functional miRNAs and coding RNAs related to cotton GMS, we used the small RNA and degradome data sequenced in our previous studies, transcriptome data newly sequenced by us, and the tetraploid upland cotton reference genome sequence to identify the miRNAs and differentially expressed genes (DEGs) during anther development between the GMS mutant and its WT cotton, which provided more information for understanding the mechanisms of cotton anther development.

## Methods

### Plant material preparation

Seeds of a genetically male-sterile cotton mutant (‘Dong A’) and its fertile WT were field grown under normal agronomic conditions at the experimental farm of the Cotton Research Institute, Chinese Academy of Agricultural Sciences. According to a previous study (Hou et al. 2002), floral buds with a longitudinal length of 5.0 mm, 6.5 mm, and 9 mm, representing the developing anthers at three developmental stages (meiosis stage, tetrad stage, and uninucleate stage, respectively), were harvested in the morning and temporarily stored on ice. Three independent biological replicates were used. The excised anthers of the GMS mutant and its WT at these three stages were frozen in liquid nitrogen and stored at  −8 0 °C for later use.

### Small RNA, degradome, and transcriptome library construction and sequencing

Total RNA was extracted from anther samples of three developmental stages from the GMS mutant and its WT using TRIzol Reagent (Invitrogen) according to the manufacturer’s instructions. The RNA quantity and quality for each sample were determined using an Agilent 2100 Bioanalyzer. Six small RNA libraries were constructed as previously described (Wei et al. 2013a, b). One cotton anther degradome library was constructed for miRNA–target identification following a method previously described (Addo-Quaye et al. 2008). The transcriptome libraries were constructed using an NEBNext Ultra RNA Library Prep kit for Illumina following the manufacturer’s instructions. Finally, the constructed libraries were sequenced using the Illumina HiSeq sequencing platform.

### MiRNA candidate identification

The raw sequences from the small RNA libraries were processed to filter out low-quality reads, poly (A) sequences, reads without a 3′-adapter or insert tag and contaminants from the adapter tags. The adapter sequences were removed from the remaining high-quality reads, and the reads larger than 30 nt or smaller than 18 nt were discarded. Then, clean reads were analyzed with a BLAST search against the Sanger Rfam database. Reads matching noncoding rRNAs, tRNAs, snRNAs, snoRNAs, and repeat RNAs were removed. The remaining clean reads were aligned to the *G. hirsutum* TM-1 genome (Zhang et al. 2015), and the mapped cotton genome sequences were retained for further miRNA analysis.

### Known and novel miRNA identification

The mapped clean reads were subjected to a BLAST analysis against known plant miRNA sequences in miRBase (https://www.mirbase.org/) to identify known miRNAs. Only sequences matched to known miRNAs with no more than one mismatch were considered known miRNAs. The rest of the mapped sequences were subsequently used for the prediction of potential novel miRNAs by the miRDeep2 program (Friedlander et al. 2012). Potential pre-miRNAs and secondary structures from the genomic sequences of *G. hirsutum* were examined. Candidate miRNAs that satisfied the gold criteria were accepted as novel miRNAs (Meyers et al. 2008). The secondary structures were further checked for free energy, dominance of the novel miRNA reads relative to other precursor-mapped small RNA reads in terms of abundance, the number of mismatches between the miRNA and the other arm of the hairpin, and no more than two asymmetric bulges in the stem region.

### Investigation of differentially expressed miRNAs

To investigate the miRNAs differentially expressed between the WT and GMS-mutant libraries, the read count of each identified miRNA was normalized to a TPM value as follows: TPM = (Read count/Mapped reads)*1,000,000. Differentially expressed miRNAs were detected using IDEG6 software (Romualdi et al. 2003). MiRNAs with a fold change (GMS/WT) ≥ 2 and a significance threshold ≤ 0.01 were considered differentially expressed miRNAs. To visualize differential expression profiles, heatmaps were constructed using R.

### MiRNA target detection by degradome sequencing

For degradome sequencing, the 20 ~ 21-nt sequences of clean full-length reads collected with degradome sequencing were matched to the *G. hirsutum* TM-1 genome for subsequent analysis. The CleaveLand pipeline (Addo-Quaye et al. 2009) was used for the detection of cleaved miRNA targets. No mismatches were allowed at the cleavage site of the 10th and 11th nucleotides of mature miRNAs. Potential miRNA targets with a *P*  < 0.05 were retained, and T-plots were created.

### DEG analysis by transcriptome sequencing

The transcriptome sequencing data were analyzed as follows: clean reads were acquired by removing reads containing adapters and low-quality reads. These clean reads were then mapped to the reference genome sequence (*G. hirsutum* TM-1) using TopHat2 software (Kim et al. 2013; Zhang et al. 2015). The mapped reads were assembled by Cufflinks software. The gene expression levels were quantified by using FPKM values. DEGs were identified using DESeq software (Anders and Huber 2010). A fold change ≥ 2 and *P*  < 0.05 were taken as the thresholds to determine whether a gene was a DEG. GO and KEGG enrichment analyses were performed to investigate the putative functions of the DEGs, as described in previous studies (Maere et al. 2005; Xie et al. 2011).

### RT-PCR

Total RNA was extracted from frozen anther samples using the Trizol method. Two micrograms of total RNA was reverse-transcribed to cDNA for miRNAs, and coding RNA genes using a TransScript miRNA First-Strand cDNA Synthesis SuperMix kit (TransGen) and SuperScript III reverse transcriptase (Invitrogen), respectively. qRT-PCRs were carried out using SYBR Premix Ex Taq (2 ×) (TaKaRa) on an ABI 7500 real-time PCR system (Applied Biosystems, Carlsbad, USA). Amplification reactions were initiated with a denaturing step of 95 °C for 5 min, followed by 40 cycles of 95 °C for 10 s and 60 °C for 30 s. Three biological replicates and three technical replicates were used for each sample. GhUBQ7 was used as an internal reference for normalization analyses. The 2^−∆∆CT^ method was used to calculate the relative gene expression levels (Schmittgen and Livak 2008). Specific primers were designed using Primer Premier 5.0 and are listed in Table S7.

## Results

### Overview of the small RNA sequencing datasets

**Fig. 1:**
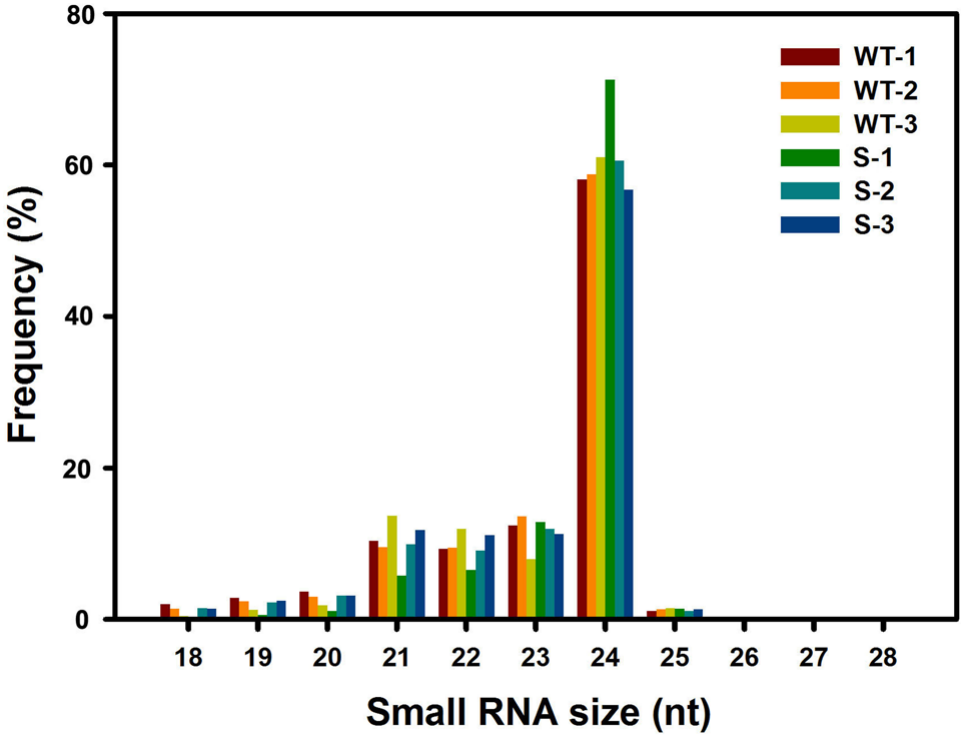
Length distribution and abundance of the small RNA sequences. WT-1, WT-2, and WT-3 represent wildtype cotton anthers at meiosis stage, tetrad stage, and uninucleate stage, respectively; S-1, S-2, and S-3 represent GMS cotton anthers at meiosis stage, tetrad stage, and uninucleate stage, respectively

High-throughput sequencing technology has greatly facilitated the pace of de novo identification of tissue- and developmental process-specific miRNAs in plants. To identify miRNAs involved in cotton anther male sterility, six small RNA libraries were constructed using the total RNA extracted from developing anthers at three developmental stages (meiosis stage, tetrad stage, and uninucleate stage) in the GMS mutant ‘Dong A’ (S-1, S-2, and S-3) and its fertile WT (WT-1, WT-2, and WT-3) and then sequenced on an Illumina HiSeq 2000 analyzer (Wei et al. 2013a, b). A total of 84.6 Mio. raw reads were obtained from the small RNA libraries, generating 13801636 (WT-1), 16421268 (WT-2), 13235815 (WT-3), 10894758 (S-1), 13166083 (S-2), and 13631786 (S-3) clean reads from the six libraries for further analysis. The unique reads from the six libraries were 8368661 (WT-1), 9434931 (WT-1), 6351108 (WT-3), 6101708 (S-1), 8145977 (S-2), and 7685111 (S-3), respectively (Table 1). Analyzing the unique reads in the libraries between the WT and its GMS mutant libraries, we found that only 1335784 (10.17%), 1802655 (11.42%), and 1409362 (11.16%) of the unique miRNAs were shared between the WT and its GMS mutant at the three anther developmental stages, respectively (Fig. S1). The size of the majority of the clean reads in the six small RNA libraries was 21–24 nt, and the 24 nt size class was the most abundant class of small RNAs, accounting for approximately 60% of the clean reads in each library (Fig. 1). These high-quality small RNA reads were analyzed with a BLAST search against the Rfam database and classified into six classes: ribosomal RNAs (rRNAs), transfer RNAs (tRNAs), small nuclear RNAs (snRNAs), small nucleolar RNAs (snoRNAs), repeats, and unannotated. The unannotated reads had the highest fraction (account for more than 95%) of total clean reads in each library, potentially including large amount of novel miRNAs, and new types of regulatory small RNAs (Table S1). Moreover, the unannotated reads were then mapped to the tetraploid upland cotton (*G. hirsutum*) TM-1 genome, generating 12098147 (WT-1; 90.22%), 14266640 (WT-2; 90.69%), 11357420 (WT-3; 89.45%), 9541401 (S-1; 88.79%), 11604711 (S-2; 90.28%), and 11678410 (S-3; 89.91%) genome-matched reads (Table 1). In each library, more than 88% of reads matched the genome sequences, indicating considerable potential to discover new regulatory small RNAs and novel miRNAs related to cotton male sterility.

**Table 1:** Summary of the small RNA sequencing data

Libraries	Raw reads	Clean reads	Uniq reads	Unannotated	Mapped genome	Ratio %
WT-1	14420272	13801636	8368661	13409815	12098147	90.22
WT-2	16953177	16421268	9434931	15732006	14266640	90.69
WT-3	13863728	13235815	6351108	12696948	11357420	89.45
S-1	11640735	10894758	6101708	10746031	9541401	88.79
S-2	13664290	13166083	8145977	12853704	11604711	90.28
S-3	14058209	13631786	7685111	12989318	11678410	89.91

### Identification of known miRNAs

To identify known miRNAs in the small RNA libraries, the genome-mapped clean reads were subjected to a BlastN search against known mature plant miRNA sequences deposited in miRBase. As a result, 80 known miRNAs belonging to 52 conserved miRNA families were identified in the six libraries. The basic information for these known cotton miRNAs in the six sRNA libraries is listed in Table S2. The expression of different miRNAs was found to be largely divergent, and their abundance ranged from 1 to 124,855 reads (Table S2). Based on the normalized transcripts per million (TPM) values, the most abundant miRNAs in our dataset were ghr-miR156a/b/c, ghr-miR166, ghr-miR167a/b, and ghr-miR3476-5p, whose TPM values were greater than 1000 in at least one library, and this result was highly consistent with those in previous studies on mosses, eudicots, and monocots (Axtell and Bartel 2005; Cui et al. 2014). However, the expression of some miRNAs, such as ghr-miR398, ghr-miR7488, ghr-miR7053, and ghr-miR827a/b/c was very low, with only several reads per library. The varied abundances of different miRNAs suggest that these miRNA genes are differentially transcribed during cotton anther development stages. In addition, the length of the identified known pre-miRNAs ranged from 74 to 463 nt, and the negative minimum free energy (MFE) of the predicted hairpins ranged from  − 52.1 to  − 97.6 kcal/mol. Of the 80 known miRNAs, 21-nt-long miRNAs were the most abundant, accounting for 41.02% of the miRNAs, followed by 24-nt miRNAs (33.33%).

### Identification of novel miRNAs

Except for the abovementioned known-miRNA mapped sequences, the genome-mapped sequences were subsequently subjected to a rigorous secondary structure analysis of their precursors and used to predict potential novel miRNAs by miRDeep2 software. A total of 220 potential novel miRNA sequences were obtained from the *G. hirsutum* genome sequences according to the gold criteria (Meyers et al. 2008), and their basic information is listed in Table S3. These novel miRNA candidates were divided into 103 families and were named temporarily in the form of nGhmiR-number before being submitted to the miRBase database. The length of these novel miRNAs ranged from 18 to 24 nt and the 21 nt long miRNAs were the most abundant (61.82%), followed by the 22 nt (14.55%) and 24 nt miRNAs (13.64%), which was consistent with the previous studies showing that miRNAs are typically 21 or 24 nt small RNAs in plants. In addition, the analysis of the first nucleotide bias in novel miRNAs showed that uracil (U), accounting for 65%, was the most prominent nucleotide at the 5′ terminus, followed by adenine (A, 22.2%), guanine (G, 7.3%), cytosine (C, 5.5%) (Table 2). This pattern is in agreement with the previous studies, mainly because AGO1 usually displays a preference for miRNAs with a 5′-terminal U (Mi et al. 2008). In addition, the MFE for the hairpin structures of the novel miRNA precursors varied from  − 25.5 to  − 166.8 kcal/mol (Fig. S2). The novel miRNAs showed a very broad range of expression levels among the six libraries, and the abundance of most novel miRNAs was very low, which was consistent with observations in other plants (Bennetzen et al. 2012). This result suggests that the majority of cotton-specific miRNAs are expressed at low levels.

**Table 2:** The 5′-terminal nucleotides and lengths of the 220 novel miRNAs

miRNA length (nt)	5′ terminal				
	A	U	C	G	Total
18	0	5	0	0	5
19	1	5	0	0	6
20	0	3	0	5	8
21	23	98	9	6	136
22	1	30	0	1	32
23	1	0	2	0	3
24	23	2	1	4	30
Total	49	143	12	16	220

### Differential expression analysis of known and novel miRNAs

**Fig. 2:**
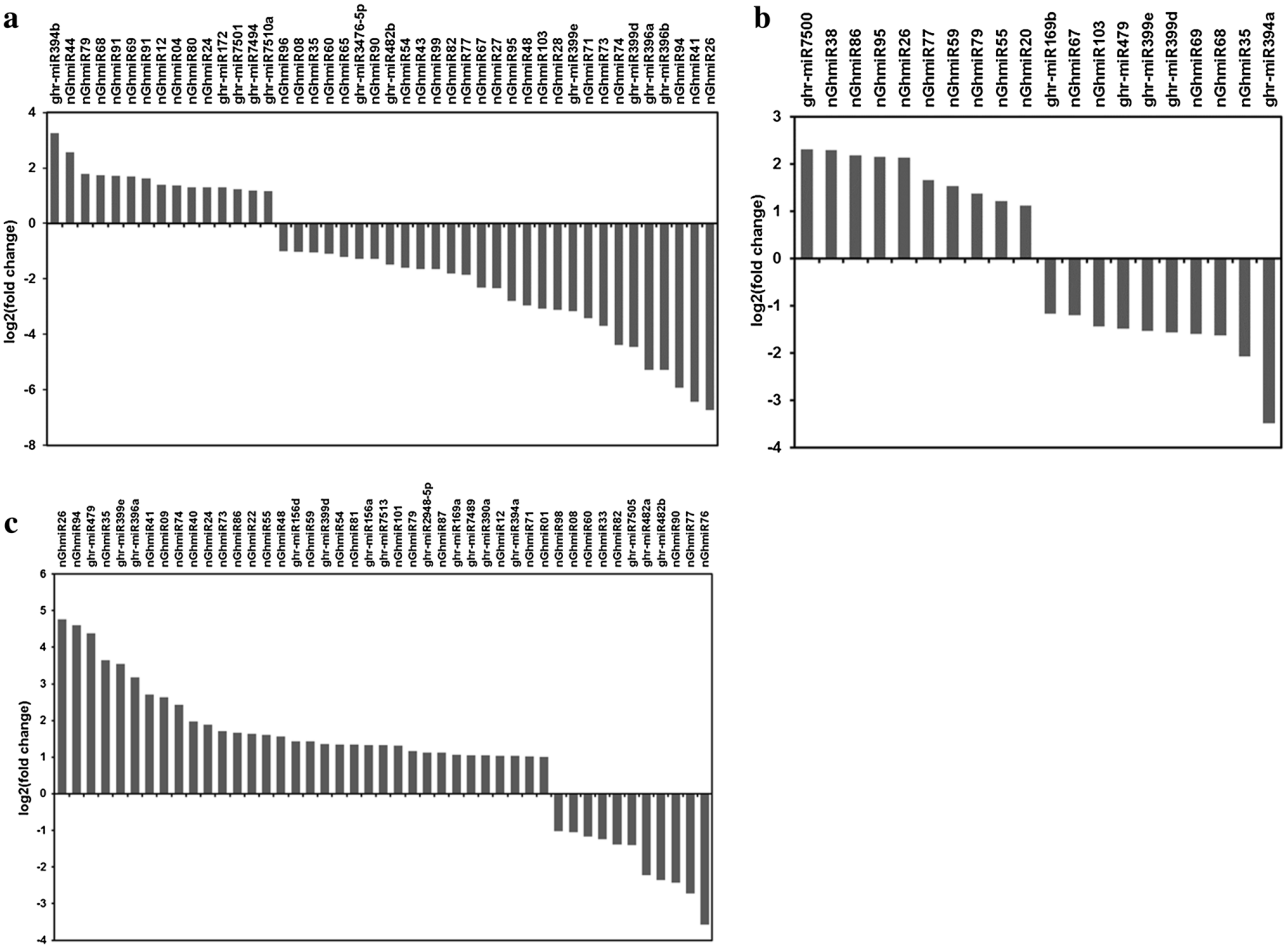
Expression profiles of differentially expressed miRNAs identified between GMS mutant and WT libraries. **a** Differentially expressed miRNAs between GMS mutant and WT cotton anthers at meiosis stage; **b** Differentially expressed miRNAs between GMS mutant and WT cotton anthers at tetrad stage; **c** Differentially expressed miRNAs between GMS mutant and WT cotton anthers at uninucleate stage

**Fig. 3:**
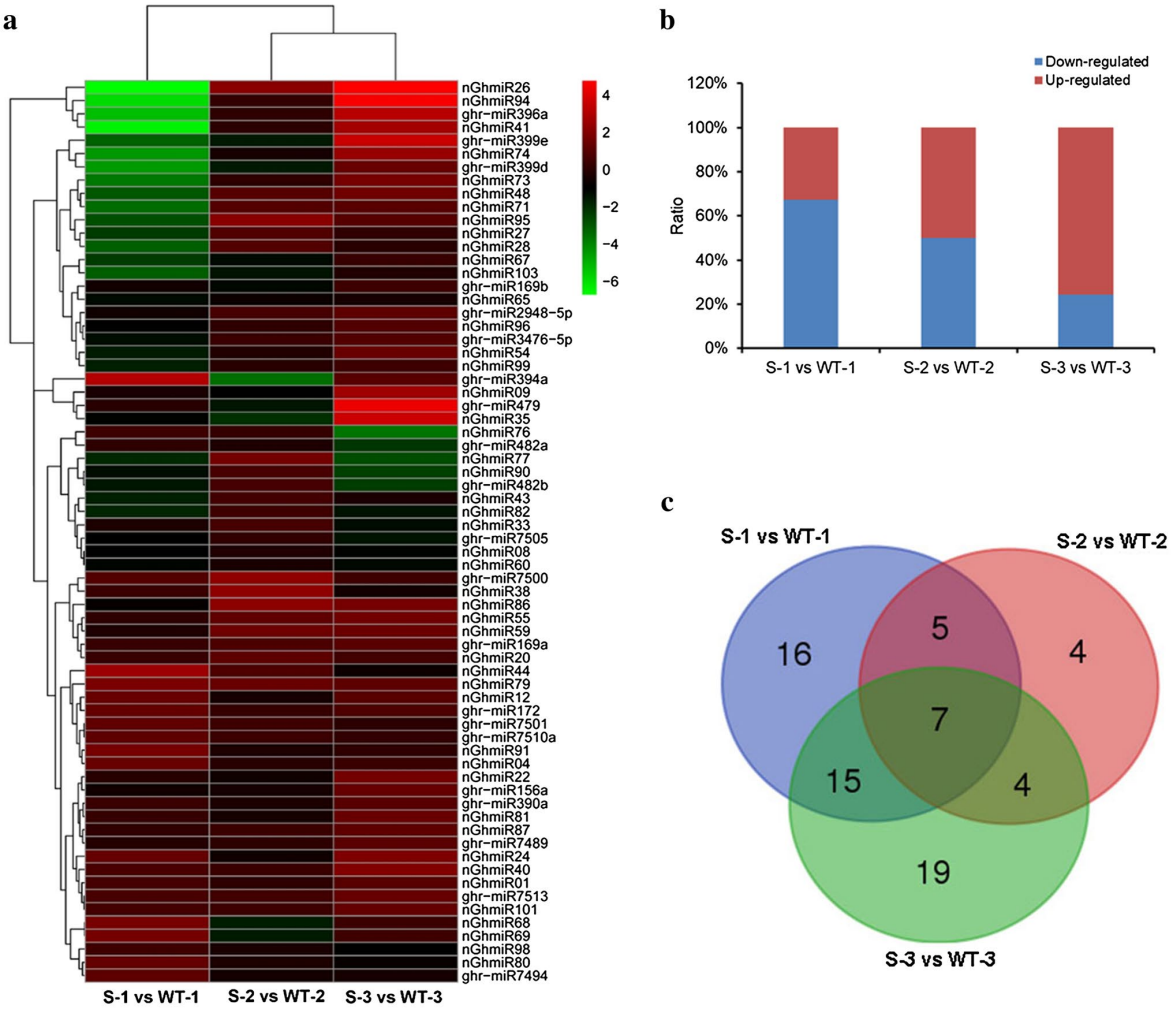
Comparative analysis of differential expression miRNAs between GMS mutant and WT libraries. **a** A complete linkage hierarchical cluster analysis of differentially expressed miRNAs identified between GMS mutant and WT libraries; **b** The overview of differentially expressed miRNAs at three anther development stages; **c** Venn diagram showing differentially expressed miRNAs at three anther development stages. WT-1, WT-2, and WT-3 represent wildtype cotton anthers at meiosis stage, tetrad stage, and uninucleate stage, respectively; S-1, S-2, and S-3 represent GMS cotton anthers at meiosis stage, tetrad stage, and uninucleate stage, respectively

To investigate miRNAs related to cotton anther development and involved in male sterility, differential expression analysis of the 80 known and 220 novel miRNAs was performed between the GMS mutant and WT libraries. To minimize noise and improve accuracy, only the miRNAs with a TPM value greater than 5 in at least one library were used for the comparison. MiRNAs with an absolute log2 fold change ≥ 1 were considered differentially expressed miRNAs. In total, 71 miRNAs were detected with differential expression during the three stages (Table S4). Forty-three miRNAs were differentially expressed during the meiosis stage (S-1 vs WT-1); of these, 14 miRNAs were upregulated, and 29 miRNAs were downregulated (Fig. 2a). In the meiosis stage, the miRNAs with the most significant expression difference were nGhmiR26 and nGhmiR41, which were approximately 100-fold downregulated. Twenty miRNAs (10 upregulated and 10 downregulated) were differentially expressed during the tetrad stage (S-2 vs WT-2) (Fig. 2b). Forty-five miRNAs (34 upregulated and 11 downregulated) were differentially expressed during the uninucleate stage (S-3 vs WT-3) (Fig. 2c). The results showed that more differentially expressed miRNAs were detected in the meiosis and uninucleate stages than in the tetrad stage, more miRNAs were significantly downregulated in the meiosis stage than in the other two stages, and more miRNAs were upregulated in the uninucleate stage than in the other two stages (Fig. 3a, b). Seven miRNAs (ghr-miR394a, ghr-miR399d/e, nGhmiR26, nGhmiR35, nGhmiR77, and nGhmiR79) were differentially expressed across all three anther developmental stages, and 16, 4, and 19 miRNAs were differentially expressed in only the meiosis, tetrad, and uninucleate stages, respectively (Fig. 3c). Taken together, the differentially expressed miRNAs demonstrated dynamic changes in the expression levels in response to different anther developmental stages and may be considered as male-sterility related miRNAs.

### Identification of miRNA targets through degradome sequencing

**Fig. 4:**
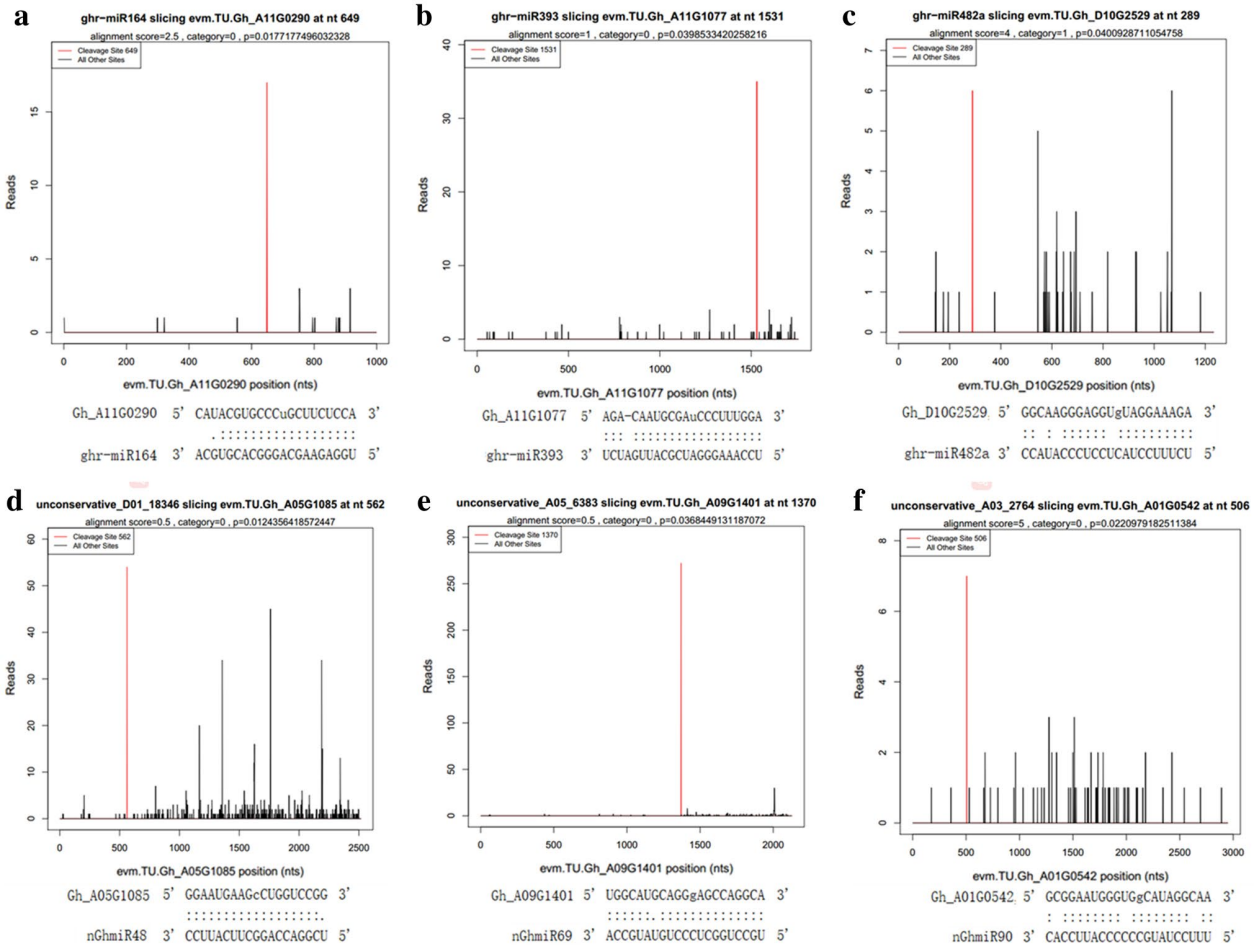
Target plots (t-plots) of the representative miRNA targets identified by degradome sequencing. Red lines indicate signatures consistent with miRNA-directed cleavage. **a** ghr-miR164 and Gh_A11G0290 (NAC domain transcription factor); **b** ghr-miR393 and Gh_A11G1077 (F-box superfamily protein); **c** ghr-miR482a and Gh_D10G2529 (P-loop nucleoside triphosphate hydrolases); **d** nGhmiR48 and Gh_A05G1085 (homeobox-leucine zipper family protein); **e** nGhmiR69 and Gh_A09G1401 (auxin response factor 16); **f** nGhmiR90 and Gh_A01G0542 (CC-NBS-LRR class family)

It is well known that miRNAs can perfectly bind to their target genes to negatively regulate gene expression by either transcript degradation or translation inhibition (Bartel 2009). Degradome sequencing is a high-throughput global experimental strategy used for accurate validation of miRNA targets, which is important in elucidating potential molecular functions of miRNA-mediated networks (Zhai et al. 2016). In this study, we employed the degradome sequencing approach to detect miRNA target genes from a library of pooled RNAs from GMS mutant and WT cotton anthers (Wei et al. 2013a, b). After removing adapter sequences and low-quality reads, 23645664 clean reads (9275280 unique reads) were acquired, 7972999 unique reads (85.96%) of which were perfectly matched to the *G. hirsutum* TM-1 genome. The mapped reads were further investigated to identify target genes using the plant-compatible pipeline software CleaveLand. A total of 117 candidate target genes were found to be cleaved by 16 known and 36 novel miRNAs (Table S5). MiRNAs were found to target various numbers of genes, with a range of 1 to 15, such as ghr-miR166b, which was identified as having only 1 targeted gene, while nGhmiR06 has 15. According to the abundance of tags at the miRNA cleavage sites, the identified targets could be classified into five classes (categories 0, 1, 2, 3, and 4), as described in a previous study (Yang et al. 2013a, b, c). Among the identified targets, the targets in category 0, which were evaluated as the most significant and reliable, were the most abundant. The representative miRNAs and corresponding target genes are presented in the form of target plots (*t*-plots), which show the distribution of the degradome tags along the full length of the target mRNA sequence (Fig. 4). Functional analysis of the identified target genes revealed that these miRNA target genes participated in various biological processes (Table S5). Most of the target genes were found to be transcription factors, such as auxin response factor (ARF), MYB, NAC domain, SBP-domain, AP2, and GRAS, which are involved in the regulation of gene expression and signal transduction; others were key genes associated with plant growth and development, such as protein kinase, transferases, ubiquitin protease, pentatricopeptide repeat (PPR) and calcium-dependent protein kinase, as well as other proteins with unclear functions. Unfortunately, the target genes of many of the known and novel miRNAs identified through small RNA sequencing were not detected in the degradome analysis.

### Transcriptome data analysis and identification of DEGs

**Fig. 5:**
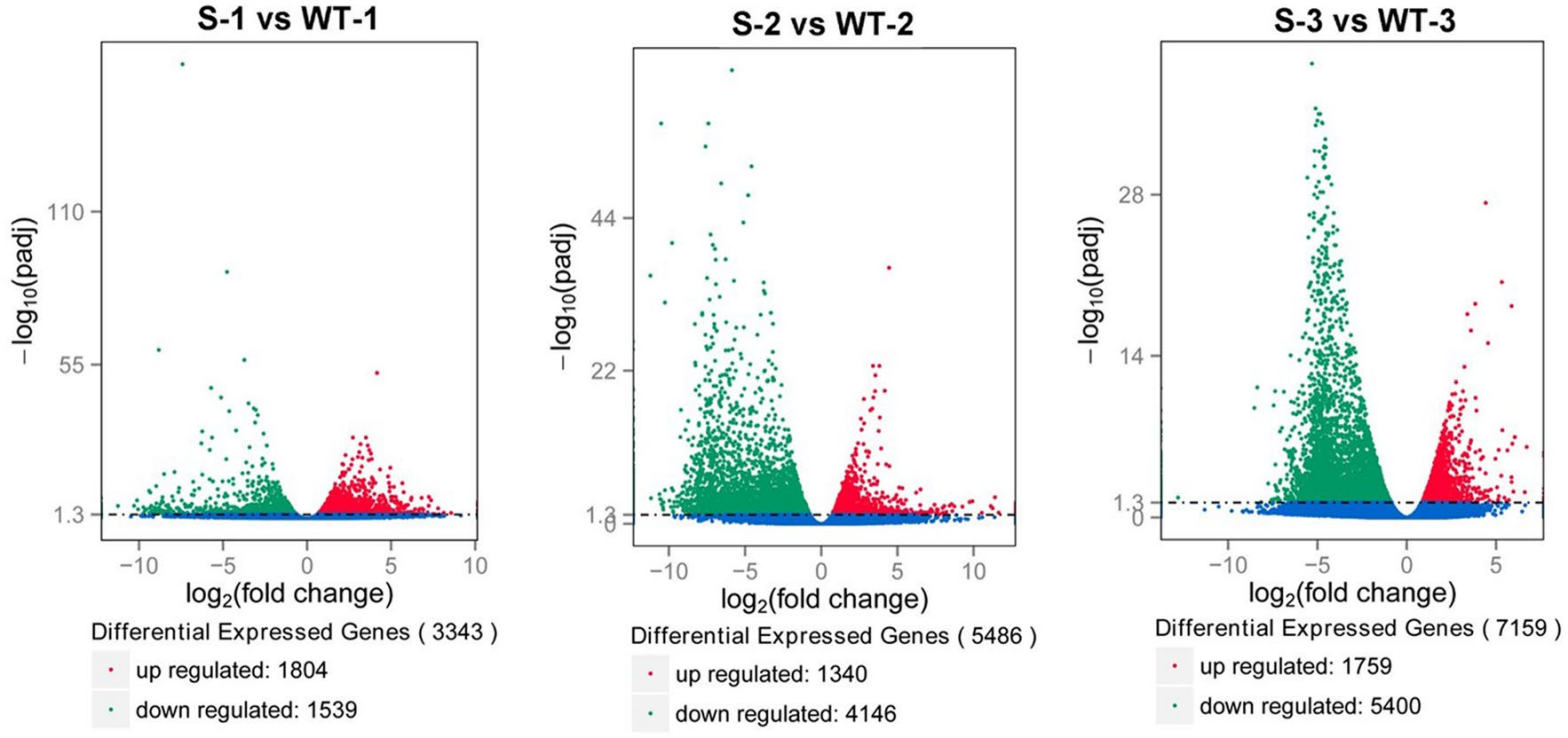
Statistical analysis of the differentially expressed genes identified by RNA-seq. Green dots indicate downregulated genes and red dots indicate upregulated genes. WT-1, WT-2, and WT-3 represent wildtype cotton anthers at meiosis stage, tetrad stage, and uninucleate stage, respectively; S-1, S-2, and S-3 represent GMS cotton anthers at meiosis stage, tetrad stage, and uninucleate stage, respectively

To characterize transcripts involved in cotton GMS, the meiosis-stage, tetrad-stage, and uninucleate-stage transcriptome libraries of the GMS mutant ‘Dong A’ and its WT were constructed, with three sample replicates for each library. These 18 libraries were sequenced by an Illumina Solexa Sequencer, which generated approximately 930 Mio. raw reads with an average length of 150 nt. After filtering out the low-quality tags, the total number of clean reads per library ranged from 41 to 58 Mio. (Table S6). For analysis purposes, the clean sequences of these libraries were aligned to the *G. hirsutum* TM-1 genome sequences. To perform differential gene expression analysis of the libraries, the fragments per kilobase of transcript per million fragments mapped (FPKM) value (mean value of three biological replicates) was used to normalize the gene expression levels. Candidate genes with a log2 fold change greater than 1 and a *P*  < 0.05 were considered DEGs. Based on this analysis, 3343 DEGs at the meiosis stage of anther development were detected between the GMS mutant and WT cotton, 1804 of which were upregulated and 1539 of which were downregulated in the GMS mutant. Similarly, 5480 DEGs, 1340 of which were upregulated and 4146 of which were downregulated, were identified to be involved in tetrad-stage GMS-mutant anthers, whereas a total of 7159 DEGs, including 1759 upregulated genes and 5400 downregulated genes, were observed in uninucleate-stage GMS-mutant anthers (Fig. 5).

To globally reveal the major functional category classification of these DEGs during cotton anther development, we conducted Gene Ontology (GO) analysis based on their corresponding biological processes, cellular components, and molecular functions. The DEGs were enriched in carbohydrate metabolic processes and hydrolase activity in the meiosis stage. The most abundant DEGs in tetrad-stage anthers were found to be enriched in cytoplasmic part, structural molecule activity, and ribonucleoprotein complex. In the uninucleate stage, most DEGs were significantly related to organonitrogen compound biosynthesis and metabolism and cytoplasm part (Fig. S3). In addition, the DEGs in the three comparison groups were further analyzed using Kyoto Encyclopedia of Genes and Genomes (KEGG) enrichment pathways. The majority of the DEGs were most likely involved in the metabolic pathway biosynthesis of secondary metabolites, starch and sucrose metabolism, ribosome, and plant hormone signal transduction. Almost all of these pathways were significantly downregulated, suggesting that they might play an important role in cotton male sterility (Table 3).

**Table 3:** KEGG enrichment analysis of the DEGs

	Total	Upregulated	Downregulated
S-1 vs WT-1			
Metabolic pathways	377	96	281
Biosynthesis of secondary metabolites	186	55	131
Starch and sucrose metabolism	95	21	74
Pentose and glucuronate interconversions	81	19	62
Protein processing in endoplasmic reticulum	53	8	45
Plant hormone signal transduction	53	15	38
Carbon metabolism	42	14	28
Biosynthesis of amino acids	39	12	27
Oxidative phosphorylation	33	6	27
Plant–pathogen interaction	33	6	27
S-2 vs WT-2			
Metabolic pathways	441	98	343
Biosynthesis of secondary metabolites	228	65	163
Ribosome	163	1	162
Starch and sucrose metabolism	101	15	86
Plant hormone signal transduction	78	29	49
Biosynthesis of amino acids	73	19	54
Pentose and glucuronate interconversions	71	4	67
Protein processing in endoplasmic reticulum	69	14	55
Carbon metabolism	65	20	45
Oxidative phosphorylation	40	4	36
S-3 vs WT-3			
Metabolic pathways	718	176	542
Biosynthesis of secondary metabolites	362	111	251
Ribosome	175	12	173
Carbon metabolism	121	17	104
Starch and sucrose metabolism	108	16	92
Plant hormone signal transduction	103	57	46
Oxidative phosphorylation	97	3	94
Biosynthesis of amino acids	97	8	89
Protein processing in endoplasmic reticulum	89	3	86
Glycolysis/gluconeogenesis	71	12	59

### qRT-PCR validation of the expression levels of miRNAs and DEGs

**Fig. 6:**
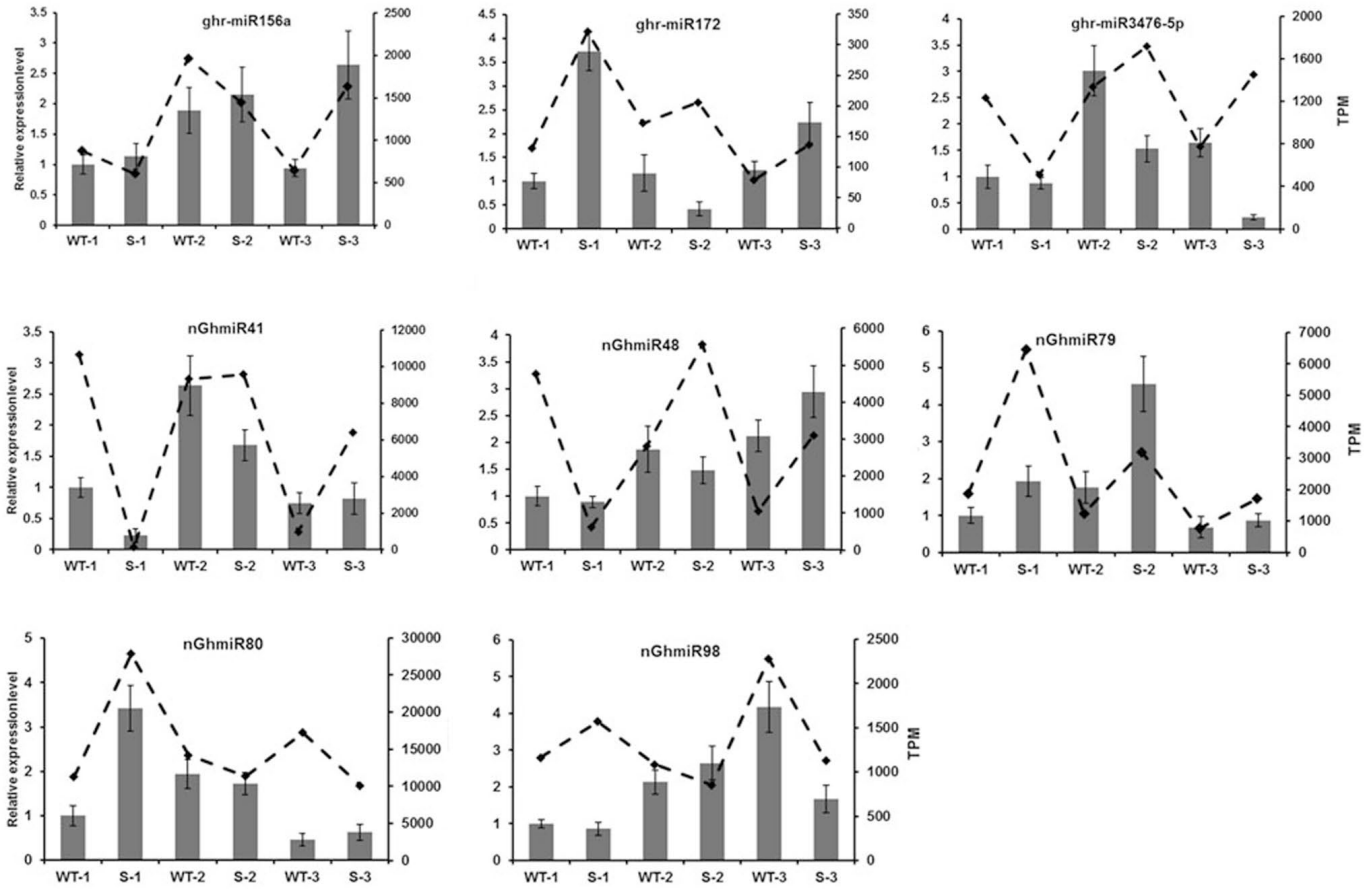
Relative expression analysis of miRNAs by qRT-PCR and small RNA sequencing. The dotted lines and bars indicate expression levels of miRNAs from small RNA sequencing and qRT-PCR results, respectively. Error bars indicate standard deviation of three biological replicates. WT-1, WT-2, and WT-3 represent wildtype cotton anthers at meiosis stage, tetrad stage, and uninucleate stage, respectively; S-1, S-2, and S-3 represent GMS cotton anthers at meiosis stage, tetrad stage, and uninucleate stage, respectively. *TPM* transcript per million clean reads

**Fig. 7:**
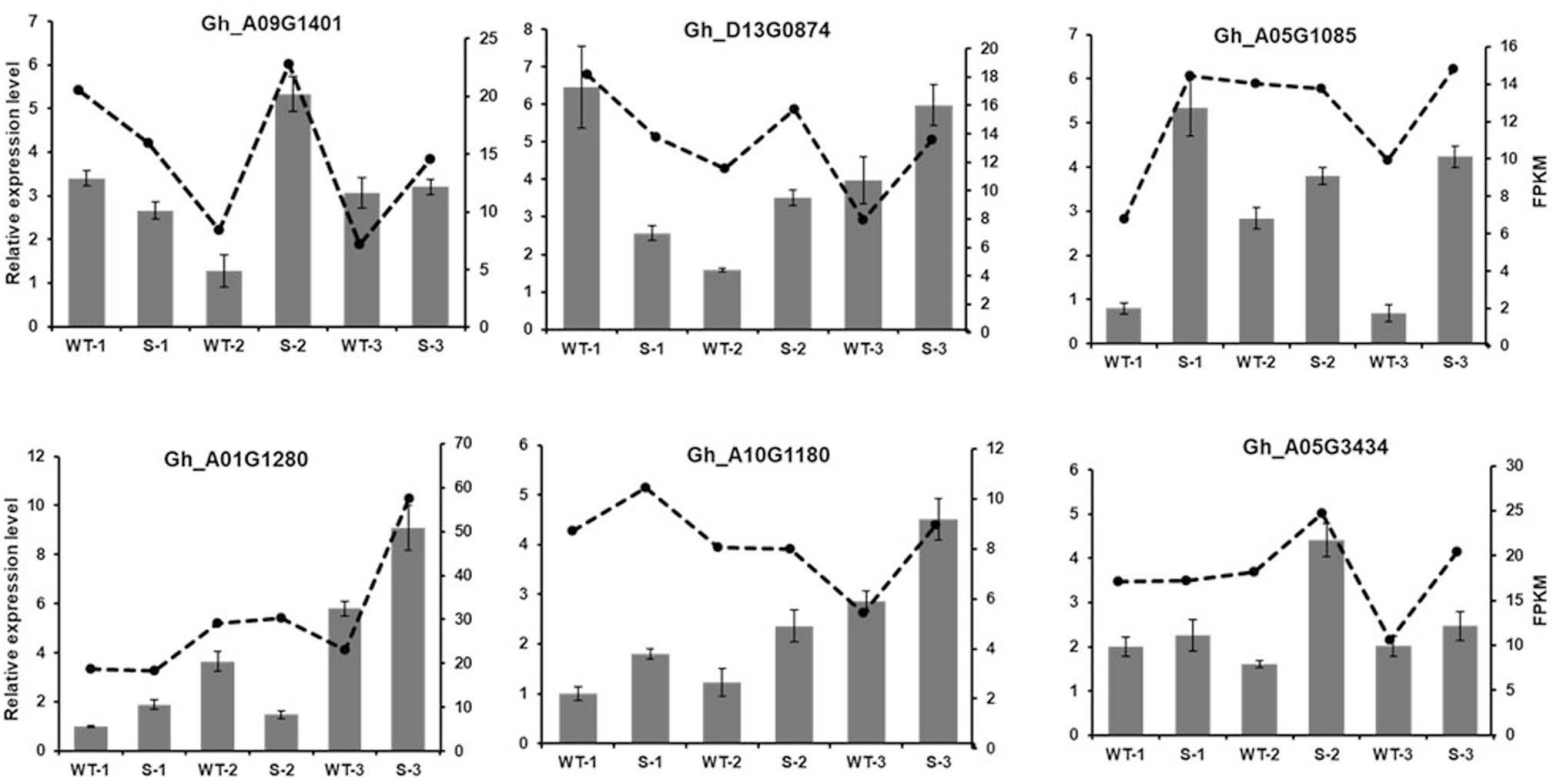
Relative expression analysis of the differentially expressed genes by qRT-PCR and RNA-seq. Dotted line shows gene expression level of FPKM, bar graph represents gene expression by qRT-PCR. Error bars indicate standard deviation of three biological replicates. Gh_A09G1401: ARF16; Gh_D13G0874: SBP transcription factor; Gh_A05G0185: SBP transcription factor; Gh_A01G1280: Galactose oxidase protein; Gh_A10G1180: P-loop nucleoside triphosphate hydrolases; Gh_A05G3434: MYB33. *FPKM* fragments per kilobase of transcript per million fragments mapped

To validate the reliability of high-throughput sequencing, qRT-PCR experiments were performed to verify the expression levels of the miRNAs and DEGs. The expression patterns of three representative known miRNAs, as well as five novel miRNAs, were compared between the GMS mutant and WT cotton anthers by qRT-PCR and small RNA sequencing (Fig. 6). The expression trends of most selected miRNAs were similar, indicating the good reliability of the small RNA sequencing technology. The expression of ghr-miR3476-5p was not consistent with that of the sequencing data, possibly because of the low expression levels in the samples and the difference in sensitivity between qRT-PCR and high-throughput sequencing technology. Furthermore, we confirmed the expression levels of six DEGs identified from RNA-seq analysis. Similar expression patterns of these six DEGs between the GMS mutant and WT cotton anthers were observed in both qRT-PCR experiments and RNA-seq based differential expression analysis (Fig. 7).

### Comprehensive analysis of the expression correlation between miRNAs and their targets

It is well known that plant miRNAs are able to suppress gene expression by transcript degradation or translational inhibition (Bartel 2009). Investigation of the expression correlation between miRNAs and their targets is important in elucidating potential biological functions of miRNAs. In this study, 53 miRNAs and 117 target genes were identified, and the expression correlation between miRNAs and their targets was measured based on the high-throughput sequencing results. For the targets predicted by degradome sequencing, 24, 11, and 21 target genes at the meiosis, tetrad, and uninucleate stages showed expression correlations with the corresponding miRNAs, respectively (Table 4). For example, the transcript levels of Gh_A01G1867 and Gh_A02G1495, the targets of ghr-miR172, were negatively correlated with the reduction in ghr-miR172 level at the meiosis stage in the GMS mutant and the WT. A similar phenomenon was observed between nGhmiR48 and its target Gh_A05G1085. Hence, these miRNAs, via their mediation of the cleavage of target mRNAs, might be critical for anther development in cotton.

**Table 4:** Comprehensive analysis of the expression correlation between miRNAs and their targets

miRNA name	TPM		Target ID	FPKM		Target annotation
	WT-1	S-1		WT-1	S-1	
ghr-miR172	131.25	321.42	Gh_A01G1867	20.27	9.40	AP2
			Gh_A02G1495	12.61	6.77	Integrase-type DNA-binding protein
ghr-miR3476-5p	1231.93	508.30	Gh_D03G1579	1.35	4.98	Unknown
ghr-miR394a	0.71	6.80	Gh_A01G1280	23.08	15.57	Galactose oxidase protein
ghr-miR482b	60.99	21.75	Gh_A06G1612	3.47	7.98	LRR and NB-ARC domain protein
			Gh_A12G0580	4.56	6.77	HOPZ-activated resistance 1
nGhmiR48	4774.37	616.35	Gh_A05G1085	13.31	35.02	Homeobox-leucine zipper family
nGhmiR60	62.77	29.22	Gh_D03G1282	7.02	12.21	AP2
			Gh_A10G0822	1.24	1.12	Integrase-type DNA-binding protein
nGhmiR65	18.90	8.15	Gh_A09G1956	89.86	115.67	Zinc knuckle (CCHC-type) family
nGhmiR68	41.73	138.63	Gh_A03G0274	19.15	10.35	ARF 10
			Gh_A05G1991	13.98	6.42	ARF 17
			Gh_A05G3576	6.18	7.21	ARF 16
nGhmiR69	43.16	138.63	Gh_A03G0274	19.15	10.35	ARF 10
nGhmiR69	43.16	138.63	Gh_A09G1401	32.10	17.79	ARF 16
			Gh_D05G3805	15.18	8.37	ARF 17
nGhmiR77	70.62	19.71	Gh_D11G2936	0.07	0.14	TIR-NBS-LRR class
nGhmiR79	1872.51	6455.00	Gh_A13G0749	29.76	12.03	SBP domain transcription factor
			Gh_A01G2095	10.19	4.71	Squamosa promoter-binding protein 9
nGhmiR80	11271.81	27876.29	Gh_A01G2095	10.19	4.71	Squamosa promoter-binding protein 9
			Gh_D13G0874	19.76	12.33	SBP domain transcription factor
nGhmiR82	33.53	9.51	Gh_D05G0081	32.90	71.27	XS domain-containing protein
nGhmiR90	36.38	14.95	Gh_A01G0542	0.43	0.33	CC-NBS-LRR class
nGhmiR99	10.70	3.40	Gh_D12G0807	7.92	8.30	Peroxidase superfamily protein

miRNA name	TPM		Target ID	FPKM		Target annotation
	WT-2	S-2		WT-2	S-2	
ghr-miR394a	4.16	0.37	Gh_D01G1502	22.22	22.95	Galactose oxidase protein
ghr-miR169b	110.91	49.65	Gh_A12G2523	30.85	17.87	TIC-like
nGhmiR68	104.16	33.60	Gh_A03G0274	5.57	9.41	ARF 10
			Gh_A05G1991	10.93	19.10	ARF 17
			Gh_A05G3576	3.20	4.64	ARF 16
nGhmiR69	105.72	35.09	Gh_A09G1401	8.45	22.79	ARF 16
			Gh_A03G0274	5.57	9.41	ARF 10
			Gh_D05G3805	11.53	23.91	ARF 17
nGhmiR77	10.91	34.34	Gh_D11G2936	0.06	0.10	TIR-NBS-LRR class
nGhmiR79	1234.56	3193.48	Gh_D13G0874	13.58	15.72	SBP domain transcription factor
			Gh_A01G2095	5.70	4.76	Squamosa promoter-binding protein 9

miRNA name	TPM		Target ID	FPKM		Target annotation
	WT-3	S-3		WT-3	S-3	
ghr-miR390a	179.98	370.60	Gh_A10G1180	5.44	8.95	P-loop nucleoside triphosphate hydrolases
ghr-miR394a	4.84	9.89	Gh_A01G1280	20.11	38.40	Galactose oxidase protein
ghr-miR482a	5.23	1.12	Gh_D10G2529	1.66	4.46	P-loop nucleoside triphosphate hydrolases
ghr-miR482b	92.22	17.99	Gh_A06G1612	4.04	8.84	LRR and NB-ARC domain
			Gh_A12G0580	5.06	6.74	HOPZ-activated resistance 1
ghr-miR7505	48.05	18.22	Gh_A10G1153	1.08	1.04	Pentatricopeptide repeat
			Gh_A10G1155	1.75	1.87	Proton gradient regulation 3
nGhmiR22	83.69	259.74	Gh_A10G2128	2.03	2.49	Protein kinase superfamily protein
nGhmiR48	1049.86	3107.63	Gh_A05G1085	9.93	14.81	Homeobox-leucine zipper family protein
nGhmiR60	66.45	29.46	Gh_D03G1282	8.13	25.56	AP2
			Gh_A02G1495	2.50	3.75	Integrase-type DNA-binding protein
nGhmiR76	26.74	2.25	Gh_D11G2531	0.18	0.03	Malectin/receptor-like protein kinase
nGhmiR77	109.46	16.64	Gh_D11G2936	0.02	0.09	TIR-NBS-LRR class
nGhmiR79	761.97	1715.39	Gh_A01G2095	4.45	3.76	Squamosa promoter-binding protein 9
			Gh_D13G0874	7.96	13.55	SBP domain transcription factor
nGhmiR82	76.53	29.23	Gh_D05G0081	21.26	47.43	XS domain-containing protein
nGhmiR90	25.38	4.72	Gh_D11G2936	0.02	0.09	TIR-NBS-LRR
			Gh_A08G2056	0.34	0.73	NAC domain-containing protein 42
			Gh_A01G0542	0.44	0.59	CC-NBS-LRR class
nGhmiR98	2279.12	1128.23	Gh_D10G1292	2.26	3.52	Pentatricopeptide repeat (PPR) protein
			Gh_A13G1041	4.75	3.21	Calcium-dependent protein kinase 32

## Discussion

### Size of small RNAs

In this study, more than 80 Mio. clean reads were obtained with small RNA sequencing. The size of the majority of the clean reads in the six small RNA libraries was 21–24 nt (Fig. 1), which is the typical size range for small RNAs and is consistent with that in previous reports on other plant species, including rice (Li et al. 2011), peanut (Zhao et al. 2010), and soybean (Song et al. 2011). More specifically, the 24-nt size class was the most abundant class of small RNAs, accounting for approximately 60% of the clean reads in each library (Fig. 1). In *Arabidopsis*, 24 nt small RNAs are mainly small interfering RNAs (siRNAs) (Lu et al. 2005). The high percentage of 24 nt small RNA sequences in cotton anther cells suggests that a large number of siRNA molecules are enriched during the anther developmental stages.

### Target on transcription factors might be a way of miRNA to regulate anther development

Previous studies have indicated that miRNAs are able to inhibit gene expression by either mRNA degradation or translation repression. Recently, degradome sequencing has provided a powerful tool for the global identification of miRNA-directed targets at the transcriptional level in plants (Addo-Quaye et al. 2008; German et al. 2008). In our study, 117 target genes were identified to be cleaved by known and novel miRNAs through degradome sequencing. A large proportion of the detected targets were transcription factors, such as ARF, MYB, NAC domain, SBP-domain, AP2, and GRAS (Table S5). Transcription factors play important regulatory roles in plant growth and development, including anther development. In *Arabidopsis*, *AtARF17* responds to auxin and plays an important regulatory role in the formation of pollen outer walls and pollen development (Yang et al. 2013a, b, c). In this study, nGhmiR68 and nGhmiR69 were identified to target *GhARF17* transcription factors based on degradome sequencing. Moreover, the expression levels of nGhmiR68 and nGhmiR69 were negatively correlated with the expression levels of *GhARF17*. This suggests that nGhmiR68 and nGhmiR69 may affect the formation of pollen walls and thus the fertility of pollen by regulating *GhARF17*. Another transcription factor, *AtMYB33*, is related to tapetum development (Millar and Gubler 2005). Tapetum development was abnormal in the *AtMYB33* mutant line, leading to degradation of pollen mother cells. In our study, the *GhMYB33* transcription factor was identified as the target gene of nGhmiR92; thus, nGhmiR92 may regulate anther development by negatively regulating *GhMYB33*. Taken together, these results suggest that these miRNAs and their targets play important roles in anther development and male sterility.

### The abnormal metabolisms of starch and sucrose may be an important factor leading to pollen abortion

Anabolism of sucrose and starch is important for anther development, and large reserves of sucrose are required to provide energy for the early stage of anther development (Oliver et al. 2007). During the late stage of anther development, the maturation of pollen grains requires the accumulation of a large amount of starch to support the germination of pollen grains (Datta et al. 2002; Mamun et al. 2006). Therefore, the disruption of sugar metabolism will seriously affect anther development and lead to male sterility. In this study, a large number of DEGs identified by transcriptome sequencing were significantly enriched in the starch and sucrose metabolic pathways (Fig. S3 and Table 3). Most of these genes involved in starch and sucrose metabolism were downregulated in the GMS mutant when compared with the WT, indicating that starch and sucrose metabolism were abnormal during anther development in the GMS mutant, which may be an important factor leading to pollen abortion. In addition, the expression levels of carbohydrate metabolism-related genes were found to be downregulated in the GMS mutant, which may lead to the obstruction of the glucose metabolism pathway during anther development in this mutant. Furthermore, plant hormones are important signaling molecules that regulate anther development. A series of plant hormones, such as ethylene and gibberellin (GA), play key roles in the regulation of anther development in plants (Thornsberry et al. 2001; Kovaleva et al. 2011). In our transcriptome study, GO and KEGG enrichment analysis revealed that the DEGs were significantly enriched in plant hormone signal transduction pathways. The expression pattern of hormone signal transduction-related genes in the GMS mutant differed from that in the WT during anther development, indicating that plant hormone signal transduction might play important roles in cotton anther development. These results suggest that starch and sucrose metabolism and plant hormone signal transduction pathways might be involved in the regulation of anther development.

In summary, a total of 300 miRNAs were identified through the high-throughput sequencing of developmental cotton anthers in a GMS mutant (‘Dong A’) and its fertile wildtype (WT). A complete comparative analysis revealed that 71 miRNAs were differentially expressed during anther development. Further, degradome sequencing identified 117 target genes cleaved by 16 known and 36 novel miRNAs. Subsequently, a large number of differentially expressed genes were identified by RNA-seq, most of which were involved in sucrose and starch metabolism, carbohydrate metabolism, and plant hormone signal transduction. In addition, the expression patterns of some target genes were validated negative correlation with the expression levels of their corresponding miRNAs. Thus, our findings might provide a valuable foundation for exploring miRNA-mediated gene regulatory networks involved in cotton male sterility.

## Electronic supplementary material

Below is the link to the electronic supplementary material.

Supplementary file1 (PDF 457 kb)

Supplementary file2 (XLSX 104 kb)
