# Supplementary material for: Identification and profiling of microRNAs and differentially expressed genes during anther development between a genetic male-sterile mutant and its wildtype cotton via high-throughput RNA sequencing
Source: Mol Genet Genomics. 2020 Mar 14;295(3):645–60. doi: 10.1007/s00438-020-01656-y (PMC7203095; doi:10.1007/s00438-020-01656-y)

Figure S1. Distribution of unique reads among the six small RNA libraries.

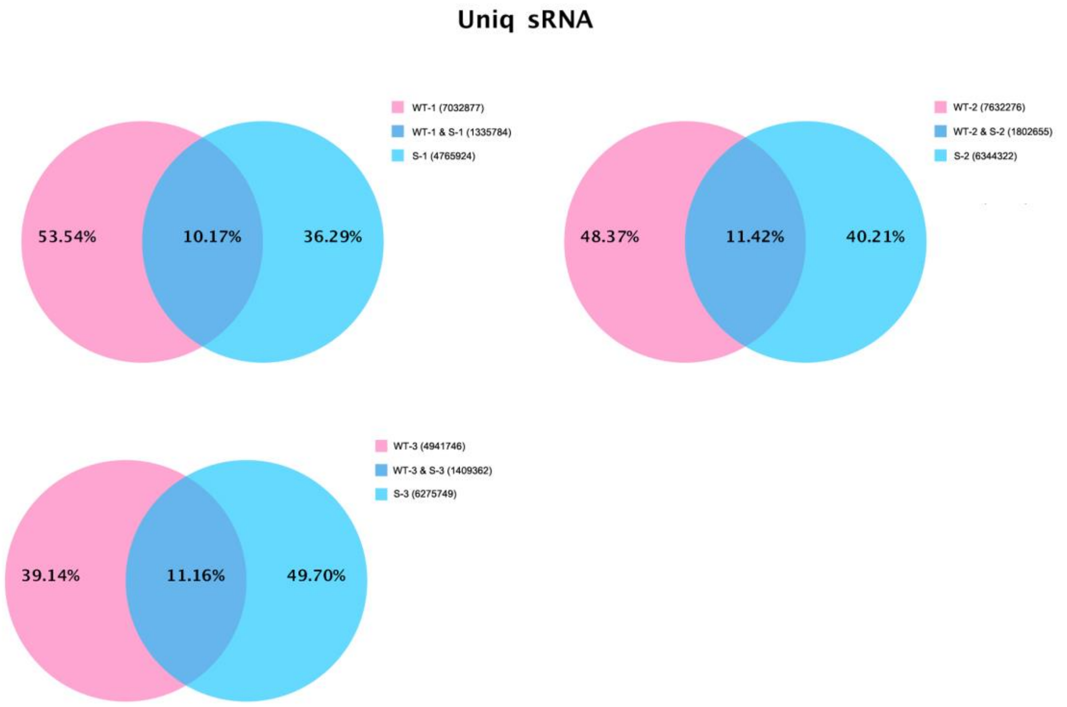

Figure S2. The secondary structures of representative novel pre-miRNAs.

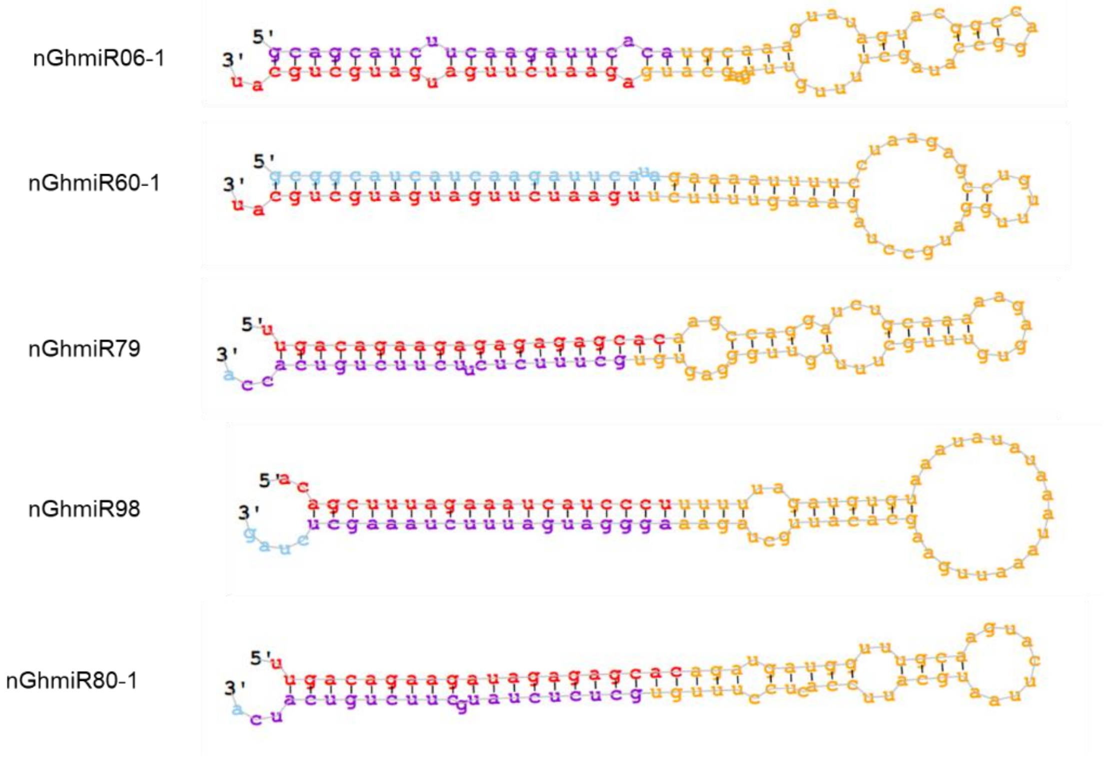

Figure S3. GO enrichment analysis of DEGs identified from RNA-seq.

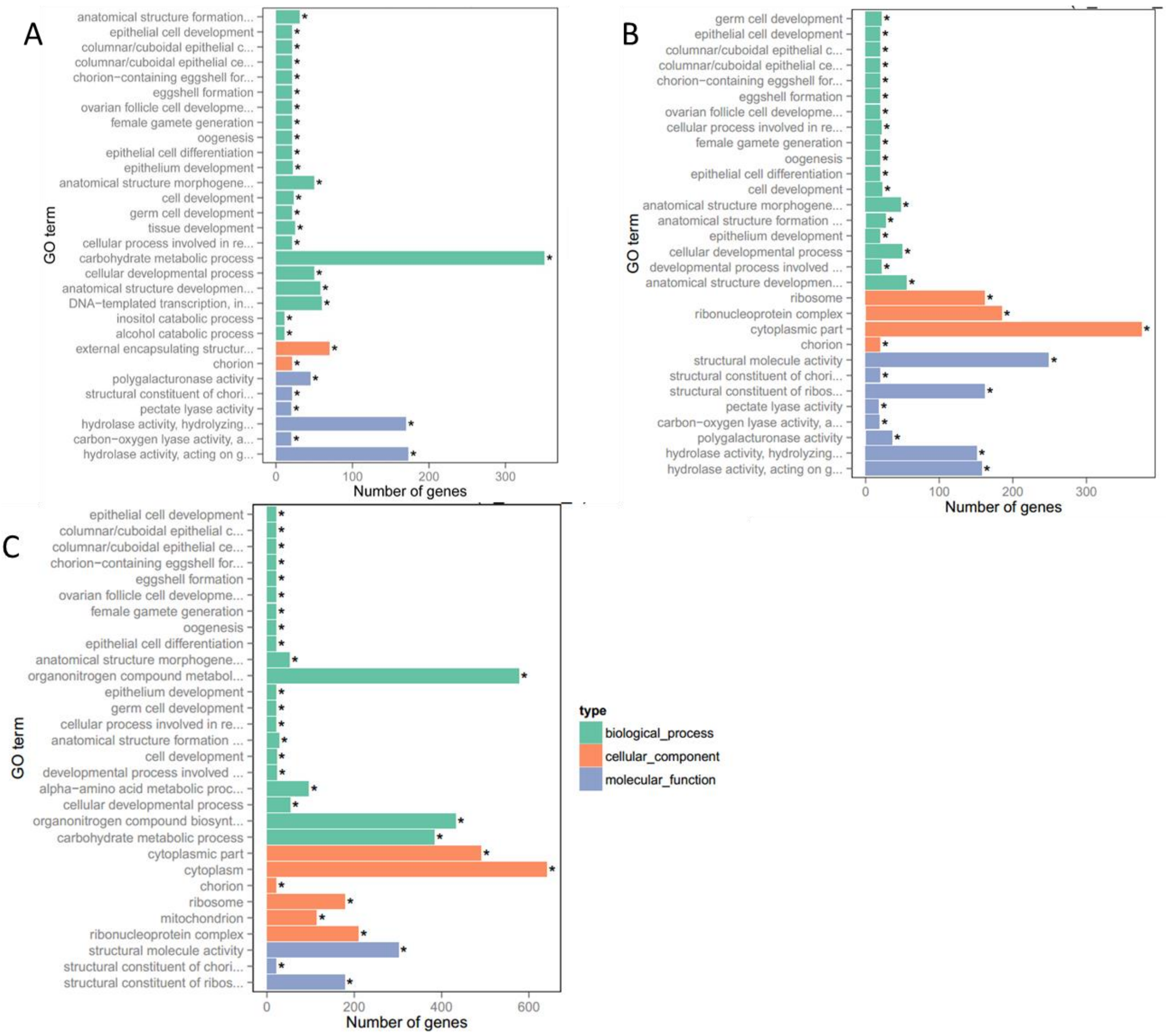

Supplement: Supplementary file 1 — Supplementary file1 (PDF 457 kb) [file 438_2020_1656_MOESM1_ESM.pdf]
